# N-acetylcysteine in substance use disorder: a lesson from preclinical and clinical research

**DOI:** 10.1007/s43440-021-00283-7

**Published:** 2021-06-06

**Authors:** Irena Smaga, Małgorzata Frankowska, Małgorzata Filip

**Affiliations:** Maj Institute of Pharmacology Polish Academy of Sciences, Department of Drug Addiction Pharmacology, Smętna 12, 31-343 Kraków, Poland

**Keywords:** N-acetylcysteine, Psychostimulant, Opioid, Cannabinoid, Nicotine, Alcohol

## Abstract

Substance use disorder (SUD) is a chronic brain condition, with compulsive and uncontrollable drug-seeking that leads to long-lasting and harmful consequences. The factors contributing to the development of SUD, as well as its treatment settings, are not fully understood. Alterations in brain glutamate homeostasis in humans and animals implicate a key role of this neurotransmitter in SUD, while the modulation of glutamate transporters has been pointed as a new strategy to diminish the excitatory glutamatergic transmission observed after drugs of abuse. N-acetylcysteine (NAC), known as a safe mucolytic agent, is involved in the regulation of this system and may be taken into account as a novel pharmacotherapy for SUD. In this paper, we summarize the current knowledge on the ability of NAC to reduce drug-seeking behavior induced by psychostimulants, opioids, cannabinoids, nicotine, and alcohol in animals and humans. Preclinical studies showed a beneficial effect in animal models of SUD, while the clinical efficacy of NAC has not been fully established. In summary, NAC will be a small add-on to usual treatment and/or psychotherapy for SUD, however, further studies are required.

## Introduction

Substance use disorder (SUD) is a chronic brain condition, with compulsive and uncontrollable drug-seeking that leads to long-lasting and harmful consequences. SUD also evokes relapse that is triggered after (re)exposure to the drug, drug-associated cues or stressors [1]. As far as public health and safety are concerned, SUD remains an unsolved issue. Several drugs can produce addictive behavior in humans and animals, including psychostimulants, opioids, cannabinoids, nicotine, and alcohol. The factors contributing to the development of SUD, as well as its treatment settings, are not fully understood.

Although the primary behavioral outcomes of addictive substances are realized through distinct effector mechanisms, such as neurotransmitter transporters, ion channels, and receptor proteins, the common feature of these drugs is concerned with increased dopamine neurotransmission within the mesocorticolimbic circuitry of the brain from the ventral tegmental area to the nucleus accumbens and prefrontal cortex [1].

Literature studies indicate that neuroadaptations within prefrontal cortical-hippocampal-striatal circuits, interconnected via glutamatergic signaling, are dysfunctional in SUD and may represent common mechanisms triggered in the case of chronic use of drugs of abuse and relapse [2]. In fact, preclinical research demonstrates that repeated exposure to several addictive substances evokes a drop in basal concentrations of extracellular glutamate [3–5]. The above change is associated with long-lasting decreases in the expression of the glial glutamate transporter 1 (GLT-1) and the cystine-glutamate exchange system/antiporter (system x_c_^−^) within the nucleus accumbens and/or prefrontal cortex related to downregulation of the genes encoding the latter system [6–11]. The system x_c_^−^ is a glycoprotein-associated amino acid transporter that catalyzes Na^+^-independent exchange of extracellular cystine for intracellular glutamate in a 1:1 stoichiometric ratio [12], and in the brain is functionally expressed as a heterodimer [13]; its catalytic unit is called xCT. Further, it influences glutamate neurotransmission by maintaining the basal level of extracellular glutamate followed by local stimulation of group II metabotropic glutamate autoreceptors. GLT-1 (also known as EAAT2) is responsible for the largest proportion of glutamate transport and control over glutamate clearance. Furthermore, in rodents drug-seeking reinstatement raises extracellular accumbal glutamate levels [14]. In line with animal research, clinical data using tailored proton magnetic resonance spectroscopy demonstrated that people with cocaine use disorder have reduced basal glutamate concentrations and increased glutamate levels in the nucleus accumbens during cue-induced craving [15]. This finding is supported by another proton magnetic resonance spectroscopy trial involving the dorsal anterior cingulate cortex, where glutamate levels were significantly higher in cocaine-dependent patients compared with healthy controls [16]. Alterations in brain glutamate homeostasis in humans and animals implicate a key role of this neurotransmitter in SUD. Additionally, the modulation of cystine-glutamate exchange via the system x_c_^−^ has been suggested as a new strategy to diminish the excitatory glutamatergic transmission observed after drugs of abuse, while drugs that influence that system—such as N-acetylcysteine (NAC)—were considered as promising targets for the development of novel pharmacotherapies of SUD.

## NAC

**Fig. 1:**
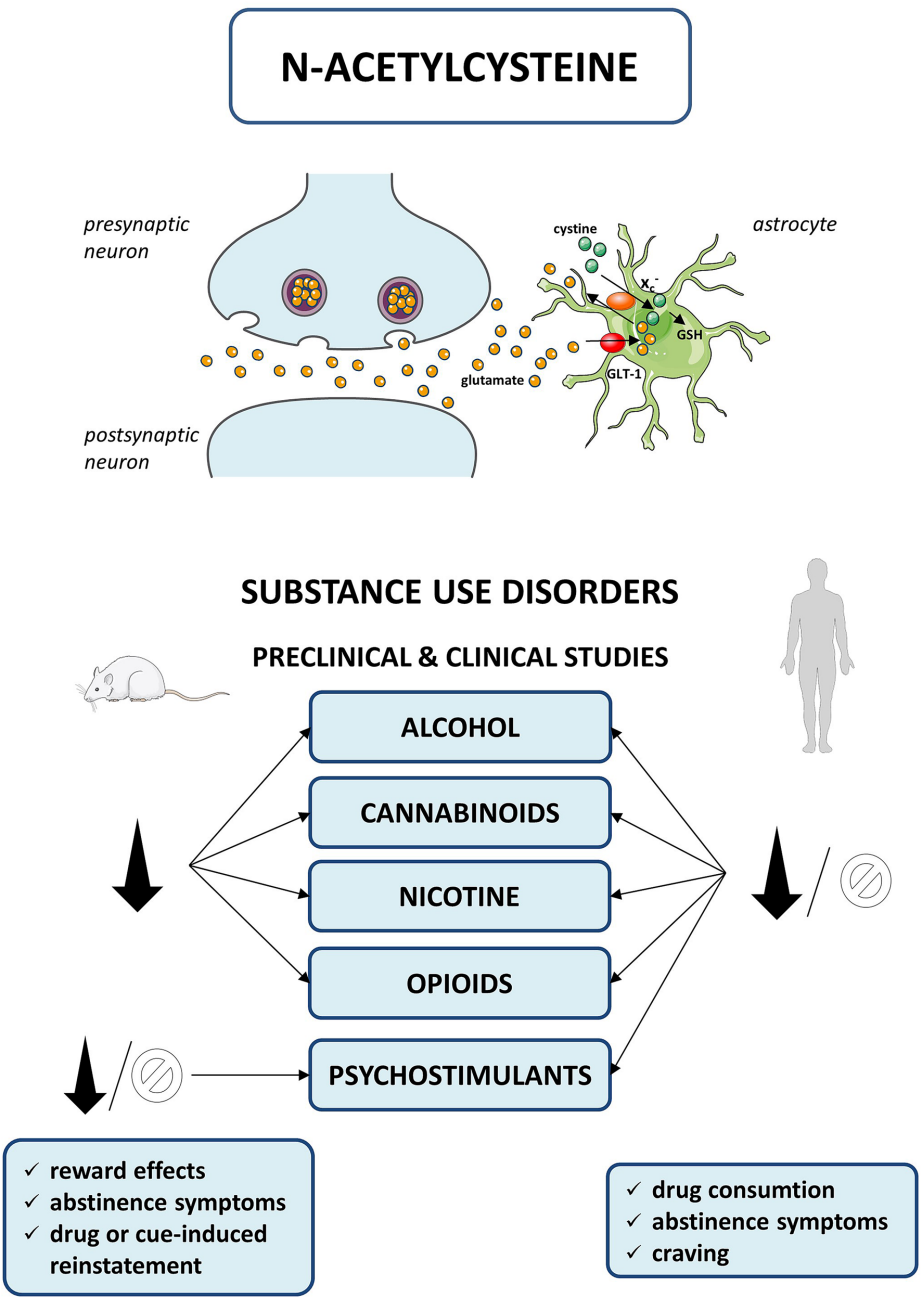
Mechanism of action and summary of N-acetylcysteine effects in preclinical and clinical studies. ∅ no changed, ↓ decreased, *GLT-1* glutamate transporter 1, *GSH* glutathione, *system x*_*c*_^*−*^ cystine/glutamate antiporter

NAC is an acetyl derivative of a semi-essential amino acid. After oral administration, NAC is rapidly absorbed from the gastrointestinal tract, and in the liver, it is transformed through deacetylation to cysteine, which (1) is used for glutathione (GSH) production, (2) enters the bloodstream and crosses the blood–brain barrier [17, 18] using a sodium-dependent transport system. However, NAC has low bioavailability in contrast to its amide derivative [19]. In the brain, cysteine is metabolized to cysteine and it modulates the synaptic release of glutamate through the plasma membrane-localized system x_c_^−^ (Fig. 1) [20]. This process activates the metabotropic glutamate receptors group II on presynaptic neurons, responsible for inhibiting the synaptic release of glutamate and thereby restoring local extracellular glutamate levels in the nucleus accumbens [10, 21]. Cysteine is the rate-limiting substrate for an important antioxidant—GSH—and, along with cystine, it also forms a key redox couple on its own. The highest plasma concentrations of NAC were detected up to 1 h following oral administration with the terminal t_1/2_ of about 6 h [22, 23].

NAC was approved for the treatment of potentially hepatotoxic doses of acetaminophen and pulmonary complications of cystic fibrosis. Apart from hepatoprotective, mucolytic, antioxidant, and anti-inflammatory activities, it has been shown that this drug exerts neurochemical effects in SUD.

## The preclinical and clinical use of NAC

This literature review summarizes research results concerning the efficacy of NAC in SUD. In the beginning, a set of conditions of searching for all experiments on animals, as well as preclinical and clinical trials (controlled and uncontrolled clinical trials, open-label cases), reported until 2020 was developed to determine the eligibility of a study using several databases, including Web of Science, Pub Med, Medline, Clinical trials and Google. All results were obtained by searching for specific keywords, as indicated in brackets, related to treatment (N-acetylcysteine—acetylcysteine), substances (marijuana/cannabinoids—tobacco/nicotine—morphine/heroin/opioid—cocaine/amphetamine/methamphetamine—alcohol) and their results (efficacy—effectiveness—outcomes—evaluation). Additionally, preclinical and clinical search terms were combined with the type of animal model (self-administration—conditioned place preference) or disorders (substance use disorders—addiction—substance abuse—dependence). Recent reviews have become increasingly focused on the potential clinical use of NAC [24, 25] in patients suffering from several psychiatric disorders, including SUD. However, this manuscript will provide a brief outline of the potential role of NAC in pharmacotherapy and examine all preclinical and clinical studies on NAC in the treatment of SUD.

### NAC and preclinical research

The first study showing the beneficial effects of NAC in SUD was published in 2003 [10]. Findings from Dr. Peter Kalivas’s laboratory indicate that systemic NAC treatment in rats evoked a dose-dependent decrease in the reinstatement of cocaine-seeking that was linked to restoring accumbal extracellular glutamate and the x_c_^−^ system [10]. From that time more than 40 research papers aimed to determine whether NAC alters reward, abstinence or reinstatement of drugs of abuse and whether the restoration of the x_c_^−^ system is accompanied by such changes were released (Table 1).

**Table 1:** NAC and SUD—preclinical research

Model/procedure	Species, sex	NAC (dose, route, treatment)	Change		References
			In vivo	Ex vivo	
Alcohol					
Chronic alcohol exposure	Wistar rats, male	2 g/L *po*; 45 day	↓ alcohol intake	↓ TG rise (serum) ↓ VLDL rise (serum) ↓ ox-LDL rise (serum) ↑ HDL/TG ratio (serum)	[26]
Chronic alcohol exposure/deprivation	Wistar rats, male	2 g/L *po*; 15 days during deprivation		↓ TG rise (serum)	
Chronic alcohol/withdrawal	Wistar rats, male	60, 90 mg/kg, *ip*	↓ alcohol withdrawal-induced anxiety (60–90)	↓ CORT rise (serum) ↓ leptin rise (serum)	[32]
Repeated alcohol in sensitization paradigm	Swiss mice, male	60, 120 mg/kg, *ip*; 15 days	↓ development of alcohol sensitization (120)	↓ ΔFosB rise (PFC) ↑ xCT drop (NAcc)	[29]
Chronic alcohol access to high alcohol drinkers/deprivation/relapse	Wistar rats, male	100 mg/kg, *po*; 14 days	↓ alcohol intake ↓ alcohol relapse	↓ GSSG/GSH and GFAP rises (HIP)	[27]
Alcohol self-administration/seeking/reacquisition	Long Evans rats, male	25, 50, 100 mg/kg, *ip*	↓ alcohol self-administration (25–100) ↓ alcohol seeking (25–100) ↓ reacquisition of alcohol self-administration (25–100)		[30]
Chronic (15 days) voluntary alcohol drinking	Sprague–Dawley rats, male	25, 50, 100 mg/kg, *ip*; 3 days	↓ depression (50–100)	↑ 5-HT drop (25–100; HIP) ↑ 5-HT drop (100; PFC) ↓ *GRIN2A* and *GRIN2B* rise (50–100; HIP) ↓ SGPT, SGOT, GGT rises (50–100) ↓ ALP, MCV rises (50–100)	[33]
Chronic (10 week) intermittent alcohol vapor/extinction/reacquisition	Wistar rats, male	25, 50, 100 mg/kg, *ip*	↓ alcohol reward (25–100) ↓ alcohol motivation (25) ↓ extinction responding (50) ↓ reacquisition of alcohol self-administration (50)	∅ xCT (100; NAc) ∅ GLT-1 (100; NAc)	[30]
Chronic alcohol access to high alcohol consumers/deprivation/relapse	Wistar rats, female	70 mg/kg, *ip*; 2 days + 40 mg/kg, *po*; 11 days	↓ alcohol intake ↓ alcohol relapse	↑ Nrf2-ARE pathway (HIP) ↓ GSSG/GSH (HIP)	[28]
Alcohol deprivation effect (ADE) model	Wistar rats, male	2 mg/h, *sc* (pumps); 14 days	↓ alcohol relapse		[31]
		60–100 mg/kg, *sc*	↓ alcohol relapse (60)		
Cannabinoids					
THC + CBD self-administration/extinction/reinstatement	Sprague–Dawley rats, male	60 mg/kg, *ip*; 5 days	↓ THC induced- reinstatement		[34]
Nicotine					
Nicotine self-administration	Wistar rats, male	30, 60, and 90 mg/kg, *ip*	↓ nicotine reward (30–90)		[37]
Nicotine self-administration		60 mg/kg; *ip*; 14 days	↓ nicotine reward (no tolerance)		
Nicotine self-administration/extinction/reinstatement		30, 60, 90 mg/kg, *ip*	↓ cue-induced reinstatement (60–90)		
Nicotine conditional place preference	ICR mice, male	5, 15, 30, 60 mg/kg, *ip*	↓ nicotine reward (5–60)		[36]
2-week continuous nicotine treatment/withdrawal		15, 30, 120 mg/kg, *ip*	↓ nicotine withdrawal (15–120) ∅ withdrawal-induced anxiety (15–120)		
Neonatal vHIP lesion + nicotine self-administration	Sprague–Dawley rats, male	100 mg/kg, *ip*; PND 42-PND 91	↓ nicotine reward		[35]
Neonatal vHIP lesion + nicotine self-administration, extinction/reinstatement		100 mg/kg, *ip;* PND 42-PND 126	↓ nicotine-induced reinstatement		
Nicotine vs. saccharin discrimination/extinction/reinstatement	Wistar rats, male	30, 60, 100 mg/kg, *ip*	↓ cue-induced reinstatement (100)		[39]
Nicotine conditioned place preference	Wistar rats, female bred as high ethanol drinkers	100 mg/kg, *po*; 9 days	↓ nicotine intake		[27]
Nicotine conditioned place preference/reinstatement			↓ reinstatement of nicotine place preference	↓ GSSG/GSH and GFAP rises (HIP)	
Nicotine self-administration/extinction/reinstatement	Sprague–Dawley rats, male	100 mg/kg *ip*; 4 days	∅ cue-induced reinstatement	∅ dendritic spine morphology rised or mRNA/protein of relevant glutamatergic genes rises (NAcc core)	[42]
		100 mg/kg *ip*; 15 days	↓ extinction ↓ cue-induced reinstatement		
Nicotine self-administration/extinction/reinstatement	Sprague–Dawley rats, male	100 mg/kg *ip*; 4 days	↓ cue-induced reinstatement		[41]
	Sprague–Dawley rats, female		∅ cue-induced reinstatement (during estrous or met/diestrus)		
Nicotine self-administration/extinction/reinstatement	Sprague–Dawley rats, male	60, 100 mg/kg *ip*; 14 days	↓ cue-induced reinstatement (100)		[40]
Nicotine self-administration/cue-exposure extinction/reinstatement			↓ cue-induced nicotine reinstatement (100)	↑ GLT-1 drop and ↓ GluN2B rise (NAcc shell; 7 days after) ↑ xCT drop (NAcc shell; 50 days after) ↑ mGluR2 (NAcc shell + core; 50 days after)	
Nicotine self-administration/home abstinence/relapse			∅ cue-induced nicotine relapse (100)		
Nicotine self-administration/extinction/reinstatement	Sprague–Dawley rats, male	100 mg/kg, *ip*; 5 days	↓ cue-induced nicotine reinstatement (100)	↓ AMPA/NMDA ratio, ↓ TNFα and ↑ GFAP (NAcc core)	[38]
Opioids					
Heroin self-administration/extinction/reinstatement	Sprague–Dawley rats, male	100 mg/kg, *ip*; 15 days	↓ cue-induced reinstatement ↓ heroin-induced reinstatement		[44]
Heroin self-administration/extinction/reinstatement	Lister Hooded rats, male	30, 60, 90 mg/kg, *ip*	∅ early cue-induced reinstatement ↓ late cue-induced reinstatement (90)		[43]
Repeated, systemic morphine administration + naloxone-precipitated withdrawal	Swiss–Webster mice, male	50 mg/kg, *ip*	↓ withdrawal symptoms (in combination with Nigella sativa oil)	↓ NO (brain) ↑ GSH (brain)	[45]
			↓ withdrawal symptoms (in combination with α-lipic acid)	↓ NO, GLU, MDA ↑ GSH, GSH-Px	[46]
Psychostimulants					
Amphetamine self-administration	Squirrel monkey, male	10, 30 mg/kg, *im*	∅ amphetamine reward ∅ cocaine reward ∅ cocaine reinstatement		[47]
Repeated amphetamine in sensitization paradigm	Sprague–Dawley rats, male	90 mg/kg, *ip*; 1 or 10 days	∅ re-expression of amphetamine sensitization		[48]
Cocaine self-administration/extinction/reinstatement	Sprague–Dawley rats, male	60 mg/kg, *ip*; 4 days	↓ cocaine-induced reinstatement	↓ GLU release rise and ↑ xCT activity drop (NAcc)	[10]
Cocaine self-administration/extinction/reinstatement	Sprague–Dawley rats, male	60 mg/kg, *sc*	↓ cocaine-induced reinstatement		[50]
Cocaine self-administration	Sprague–Dawley rats, male	60 mg/kg, *ip*; 11 days	∅ cocaine reward acqusition ∅ cocaine reward		[21]
Cocaine self-administration in escalation paradigm		60 mg/kg, *ip*; 11 days	↓ cocaine-induced escalation of drug intake		
Cocaine self-administration/extinction/reinstatement		60 mg/kg, *ip*; 10–11 days	↓ cocaine-induced reinstatement	↑ xCT and basal GLU (NAcc) ↓ cocaine-evoked GLU release (NAcc)	
Repeated cocaine in sensitization paradigm		60 mg/kg, *ip*; 7 days	↓ cocaine-induced development of behavioral sensitization		
Cocaine self-administration in extended paradigm/extinction/reinstatement	Sprague–Dawley rats, male	30, 60 mg/kg, *ip*	↓ cocaine-induced reinstatement (30, 60)		[14]
Cocaine self-administration/extinction/reinstatement		90 mg/kg, *ip*; 12 days (acquisition of self-administration)	↓ cocaine-induced reinstatement		
Cocaine self-administration/extinction/reinstatement	Sprague–Dawley rats, male	33, 100 mg/kg, *ip*; 12 days	↓ cocaine-induced reinstatement (100)	↑ AMPA/NMDA ratio drop (NAcc)	[51]
Cocaine self-administration/extinction	Sprague–Dawley rats, male	100 mg/kg, *ip*; 7 days		↑ xCT and GLT-1 drop (NAcc)	[8]
Cocaine self-administration	Sprague–Dawley rats, male	60 mg/kg, *ip*; 12 days	∅ cocaine reward		[78]
Cocaine self-administration/extinction/reinstatement		60 mg/kg, *ip*; 7 days	↓ cocaine-induced reinstatement		
Cocaine self-administration/extinction/reinstatement	Sprague–Dawley rats, male	60, 100 mg/kg, *ip*; 12 days	↓ cocaine-induced reinstatement (100) ↓ cue + cocaine-induced reinstatement (100)		[52]
Cocaine self-administration/home abstinence/relapse			↓ context-induced relapse (100)		
Cocaine self-administration	Squirrel monkeys, male	10 mg/kg, *im*	∅ cocaine reward		[47]
Cocaine self-administration/extinction/reinstatement			∅ cocaine-induced reinstatement		
Cocaine self-administration/extinction/reinstatement	Sprague–Dawley rats, male	1, 10 ug/side, intra-NAcc	↓cocaine-induced reinstatement (1–10) ↓ cue + cocaine-induced reinstatement (1–10)		[53]
Cocaine self-administration/extinction/reinstatement		10 mg/kg, *ip*	∅ cocaine-induced reinstatement ↓ cocaine-induced reinstatement (in combination with MTEP)		
Cocaine self-administration	Lister Hooded rats, male	30, 60, 90 mg/kg, *ip*	∅ cocaine reward		[54]
Cocaine self-administration/extinction		15, 30, 60, 90 mg/kg, *ip*	↓ early cocaine-induced seeking (30–90)		
Cocaine self-administration	Wistar rats, male	25–100 mg/kg, *ip*	∅ cocaine reward		[55]
		100 mg/kg, *ip*; 6 days	∅ cocaine reward		
Cocaine self-administration/extinction/reinstatement		12.5–50 mg/kg, *ip*	↓ cue-induced reinstatement (12.5–50) ↓ cocaine-induced reinstatement (25–50)		
Cocaine self-administration/extinction/reinstatement	Sprague–Dawley rats, male	100 mg/kg, *ip*; 5 days	↓ cue-induced reinstatement	↑ GLT-1 drop (NAcc)	[58]
Cocaine self-administration	Sprague–Dawley rats, male	60 mg/kg, *ip*; 19 days	∅ cocaine reward	↓ Zif268 drop (NAcc, DSTR)	[57]
Cocaine self-administration in escalation paradigm			∅ cocaine reward ↑ abstinence	↑ GLT-1 drop and ↑ Zif268 drop (NAcc, DSTR)	
Cocaine self-administration/extinction/reinstatement	Wistar rats, male	100 mg/kg, *ip*; 10 days	↓ cue-induced reinstatement ↓ cocaine-induced reinstatement		[19]
Bulbectomy + cocaine self-administration/extinction/reinstatement			∅ cue-induced reinstatement ∅ cocaine-induced reinstatement		
Neonatal vHIP lesion + repeated cocaine in sensitization paradigm	Sprague–Dawley rats, male	100 mg/kg, *ip;* PND 28-PND 84	∅ development of cocaine behavioral sensitization		[35]
Cocaine self-administration	Squirrel monkeys, male	10 mg/kg, *im*; 10 days	∅ cocaine reward		[56]
Cocaine self-administration/extinction/reinstatement		10 mg/kg, *im*; 15 days	↑ extinction ∅ cocaine-induced reinstatement		
Methamphetamine self-administration/extinction/reinstatement	Sprague–Dawley rats, female	30, 60, 120 mg/kg, *ip*	∅ methamphetamine reward ∅ methamphetamine-induced reinstatement		[49]

↑ increased, ∅ no changed, ↓ decreased, *ΔFosB* protein encoded by the FBJ murine osteosarcoma viral oncogene homolog B (FOSB) gene, *5-HT* serotonin, *ALP* alkaline phosphatase, *AMPA* α-amino-3-hydroxy-5-methyl-4-isoxazole propionic acid receptor, *CBD* cannabidiol, *CORT* corticosterone,* DSTR* dorsal striatum, *GFAP* glial fibrillary acidic protein, *GGT* gamma-glutamyl transferase, *GLT-1* glial glutamate transporter 1, *GLU* glutamate, *GluN2B* NMDA receptor subunit 2B, *GRIN2A* gene encoding NMDA receptor subunit 2A, *GRIN2B* gene encoding NMDA receptor subunit 2B, *GSH* glutathione, *GSH-Px* glutathione peroxidase, *GSSG* oxidized glutathione, *HDL* high-density lipoprotein, *HIP* hippocampus, *MCV* mean corpuscular volume, *MDA* malondialdehyde, *mGluR2* metabotropic glutamate receptor type 2, *MTEP* 3-((2-Methyl-4-thiazolyl)ethynyl)pyridine, a selective allosteric antagonist of the metabotropic glutamate receptor subtype 5, *NAC* N-acetylcysteine, *NAcc* nucleus acumbens, *NMDA* N-methyl-d-aspartate receptor, *NO* nitric oxide, *Nrf2-ARE* transcription NF-E2-related factor 2 binds to antioxidant responsive element, *ox-LDL* oxidized-low-density lipoprotein, *PFC* prefrontal cortex, *PND* postnatal day, *SGOT* serum glutamic oxaloacetic transaminase, *SGPT* serum glutamic pyruvic transaminase, *TG* triacylglycerol, *THC* tetrahydrocannabinol, *TNFα* tumor necrosis factor alpha, *vHIP* ventral hippocamapus, *VLDL* very low-density lipoprotein, *xCT* cystine-glutamate antiporter, *Zif268* zinc finger protein 268

In rodent models of alcohol use disorder (AUD), NAC given in acute doses or repeatedly (during abstinence period) reduced alcohol intake [26–28], and was responsible for the development of alcohol sensitization [29], extinction responding [30], alcohol relapse [27, 28, 31], alcohol withdrawal-induced anxiety and depression [32, 33] (Table 1). These changes were examined in male rats or mice. However, a recent paper by Quintanilla et al. also demonstrated inhibitory actions of NAC towards alcohol intake or relapse in female rats [28]. Interestingly, reductions seen after NAC treatment in behavioral studies were accompanied by either restoration in the accumbal x_c_^−^ system [29] or no changes [30]. Although the changes in the x_c_^−^ system following NAC in AUC are not resolved, further examinations of glutamate-related neuroadaptations in animal alcohol models revealed a decrease in the drug-related enhanced hippocampal oxidized/reduced glutathione ratio levels in both male [27] and female [28] rats. Additionally, NAC attenuated neuroinflammation expressed by the glial fibrillary acidic protein immunohistochemistry in the rat hippocampus, showing for the first time a new target for NAC treatment [28].

NAC effects in cannabinoid use disorder were addressed in a separate paper. Thus, Spencer et al. indicated that daily treatment with NAC attenuated cue-induced reinstatement of Δ^9^-tetrahydrocannabinol and cannabidiol seeking in rats [34].

Considering nicotine use disorder (NUD), there are several data supporting beneficial effects of acute or chronic NAC treatment on nicotine reward assessed in self-administration or conditioned place preference rodent models [27, 35–37] (Table 1). Importantly, no indication of tolerance development was observed after chronic NAC treatment toward nicotine reward [37]. Furthermore, NAC blocked mouse behaviors associated with nicotine somatic withdrawal signs, but not anxiety developed during nicotine withdrawal [36]. Other proofs that NAC may have high clinical utility in NUD were provided in reports showing the drug reducing efficacy in models assessing nicotine seeking and reinstatement behaviors [35, 38–42] (Table 1). Of note, subchronic NAC administration (< 4 days) was found as ineffective in reducing cue-induced reinstatement and in restoring nicotine-evoked disruption in dendritic spine morphology and glutamatergic transcripts in the accumbal core region [42]. In other molecular and neurochemical assays combined with behavioral evaluations, it was found that NAC effectively reversed a drop in the accumbal x_c_^−^ system and GLT-1, seen 7 or 50 days after cue-induced reinstatement, respectively [39, 40]. The latter paper even reports the anti-relapse activity of NAC with cue exposure therapy that persisted 50 days after drug treatment, supporting the idea of adopting a combined strategy for treating NUD. Interestingly, NAC did not alter cue-induced reinstatement in female rats regardless of their estrous cycle phase, which may suggest NAC sex-specific efficacy and some limitation in its use [41]. However, based on a separate report, it is difficult to draw the final conclusion and more data addressing both genders are required.

Four reports addressed the effects of NAC in rodent models of opioid use disorder (Table 1). In rats extinguishing from heroin self-administration, NAC reduced cue- or drug-evoked reinstatement precipitated after 10–40 days of heroin withdrawal [43, 44]. NAC used as an add-on drug attenuated the development of morphine tolerance and dependence in mice and associated biochemical alterations, such as reduced GSH level and GSH peroxidase activity [45, 46].

Considering amphetamines use in rats and nonhuman primates (Table 1), NAC neither changed amphetamine or cocaine reward, cocaine relapse [47], re-expression of amphetamine sensitization [48], methamphetamine reward nor reinstatement [49].

On the other hand, there is no doubt that in rat models of cocaine use disorder (CUD), NAC did not change cocaine reward, but effectively reduced escalation of drug intake, cocaine-seeking, and reinstatement behaviors [8, 10, 14, 19, 21, 50–57] (Table 1). The latter effects were observed after systemic or intra-accumbens NAC administration. Furthermore, the behavioral attenuation of cocaine actions appeared together with NAC-induced restoration of the x_c_^−^ system, GLT-1, and AMPA/NMDA ratio in accumbal or striatal brain regions [8, 21, 57, 58]. Taken together, preclinical works have supported the role of imbalances in the accumbal glutamatergic system as a driver of addictive behaviors in rodents. However, in this context, it should be added that NAC did not show efficacy in monkeys toward cocaine-induced reinstatement [47]. Since people with cocaine use disorder suffer from depression, such comorbidity was evaluated using cocaine self-administration/extinction/reinstatement procedures in the rat model of depression based on the removal of the olfactory bulbs [19]. In the latter paper it was shown that repeated treatment with NAC did not alter reinstatement of cocaine-seeking behavior, while its amide derivative, that is AD4, effectively blocked cue- or cocaine-induced reinstatement [19].

To summarize, studies on male rats show that NAC is able to significantly diminish the propensity to seek drugs of abuse (Table 1). The molecular or neurochemical mechanisms underlying such NAC effects are not fully recognized as the x_c_ system was found as unnecessary to reduce cocaine-seeking [58]. New—and independent of the x_c_^−^ system—mechanisms of NAC, such as restoring GLT-1 [58] or GSH levels [27, 28] or influencing immunomodulatory markers (the nuclear factor kappa-light-chain-enhancer of activated B cells signaling pathway) [38] were proposed.

### NAC and clinical research

Apart from pre-clinical studies, also clinical trials addressed the role of NAC therapy in diminishing relapse to addictive drug use. To date, NAC demonstrated promising results in subjects with cocaine, heroin, and tobacco addiction.

About 30% effectiveness of NAC on alcohol consumption was shown in adults during cannabis cessation [59]. One study regarded co-occurrence of AUD and post-traumatic stress disorder [60]; however, further studies are required for the determination of NAC effectiveness on alcohol consumption in patients suffering from AUD (Table 2).

**Table 2:** NAC and SUD—clinical research

Study design	Study sample size and average age [year]	NAC—dosage and duration of drug intervention	Add-on therapy	Results	References
Alcohol					
Double-blind, randomized, placebo-controlled in cannabis use disorder subjects	142 NAC/135 PB Age: 18–50	1.2 g × 2 daily for 12 weeks		↓ (weak effect) alcohol consumption	[59]
Double-blind, randomized, placebo-controlled in PTSD subjects	100 NAC/100 PB Age: 18–65	2.4 g × 2 daily for 12 weeks		Ongoing study	[60]
Cannabinoids					
Open-labelled	24 Mean age: 19	1.2 g × 2 daily for 4 weeks		↓ self-reported marijuana use and craving Ø negative urine cannabinoid tests	[61]
Double-blind, randomized, placebo-controlled	58 NAC/58 PB Mean age: 18.9	2.4 g daily for 8 weeks	Contingency management and cessation counseling	↑ abstinence ↑ negative urine cannabinoid tests	[62]
	45 NAC/44 PB Age: 15–21	2.4 g daily for 8 weeks		Ø craving ↑ negative urine cannabinoid tests	[63]
Double-blind, randomized, placebo-controlled	57 NAC/58 PB Mean age: 18.9	1.2 g × 2 daily for 8 weeks		↑ abstinence ↓ impulsivity ↑ negative urine cannabinoid tests	[64]
Double-blind, randomized, placebo-controlled	153 NAC/149 PB Age: 18–50	1.2 g × 2 daily for 12 weeks	Contingency management	Ø marijuana use Ø negative urine cannabinoid tests	[65]
	depressive patients 151 NAC/151 PB Age: 18–50			Ø marijuana use Ø abstinence Ø depression Ø negative urine cannabinoid tests	[66]
Nicotine					
Double-blind, randomized, placebo-controlled	14 NAC/15 PB Age: 50 years	2.4 g daily for 4 weeks		↓ (small change) number of cigarettes smoked daily Ø craving Ø withdrawal Ø CO levels	[67]
Double-blind, randomized, placebo-controlled	10 NAC/12 PB Mean age: 20.8	3.6 g daily for 3.5 days		Less pleasure in the first cigarette smoked Ø craving Ø withdrawal	[68]
Double-blind, randomized, placebo-controlled in pathological gamblers	13 NAC/15 PB Mean age: 47.6	1.2–3 g daily for 12 weeks	Behavioral therapy	Ø change up to 6 weeks ↓ problem-gambling severity at 3 months	[71]
Double-blind, randomized, placebo-controlled	34 NAC/34 PB Mean age: 18.8	2.4 g daily for 8 weeks		Ø number of cigarettes smoked daily Ø withdrawal	[72]
Double-blind, randomized, placebo-controlled	8 NAC/8 PB Mean age: 36.5	1.2 g × 2 daily for 3.5 days		↑ abstinence ↓ craving ↑ resting-state functional connectivity in frontostriatal areas	[69]
Open-labelled	19 Age: 18–65	1.2 g × 2 daily for 4 weeks	Varenicline	↓ number of cigarettes smoked daily low abstinence score at the study end	[70]
Double-blind, randomized, placebo- controlled	17 NAC/14 PB Mean age: 51.4	3 g daily for 12 weeks		↓ number of cigarettes smoked daily ↓ depression ↓ CO levels	[73]
Double-blind, randomized, placebo-controlled parallel	60 NAC/60 PB	1.8 g daily for 16 weeks		Ongoing study	[75]
Double-blind, randomized, placebo-controlled	17 NAC/17 PB Mean age: 47	1.8 g daily for 12 weeks		↓ CO levels ↓ sTNF-R2 levels Ø withdrawal Ø depression Ø anxiety Ø blood pressure Ø glucose	[74]
Psychostimulants					
Cocaine					
Double-blind, placebo-controlled crossover	13 Mean age: 37.1	2.4 g (0.6 g daily) for 4 days		Trends to ↓ self-reported cocaine use and craving	[76]
Double-blind, placebo-controlled crossover	15 Mean age: 37.4	2.4 g (0.6 g daily) two 3-day hospitalization separated by 4 days		↓ desire to use cocaine ↓ cue associated with cocaine use Ø craving Ø physiological response	[77]
Open label	16 Mean age: 40	1.2 or 2.4 or 3.6 g daily for 4 weeks		↓ self-reported cocaine use (2.4–3.6 g)	[79]
Single blind	6 Mean age: 41.8	1.2–2.4 g daily for 4 days	Baclofen	↓ craving ↓ motivational qualities of a cocaine challenge Ø euphoric properties	[78]
Open label randomized, crossover	8 NAC-cocaine users/ 14 NAC-healthy Mean age: 35.4	2.4 g, single dose		↓ impulsivity ↓ glutamate levels in dorsal anterior cingulate cortex	[16]
Double-blind, randomized, placebo- controlled	40 NAC (1.2 g), 33 NAC (2.4 g)/38 PB Mean age: 43.2	1.2 or 2.4 g daily for 8 weeks		Ø craving Ø abstinence ↑ (weak) abstinence	[82]
Within-subjects, double-blind, crossover	14 Mean age: 42.6	2.4 g daily for 7 days		↓ cocaine intranasal self-administration ↓ incentive salience of cocaine cue	[83]
Double-blind, randomized, placebo-controlled	9 NAC/15 PB Age: 18–55	2.4 g daily for 25 days		Ø craving Ø working memory Ø self-reported abstinence ↑ cognitive control ↓ cocaine-positive urine tests	[81]
Methamphetamine					
Double-blind, randomized, placebo-controlled	14 NAC/17 PB Mean age: 36.8	0.6 up to 2.4 g for 8 weeks	Naltrexone	Ø craving Ø methamphetamine use Ø urine toxicology	[85]
Double-blind, placebo-controlled crossover	23 Mean age: 29.2	1.2 g daily for 8 weeks		↓ craving during treatment	[86]

↑ increased, Ø no changed, ↓ decreased, *CO* carbon monoxide, *NAC* N-acetycysteine, *PB* placebo, *sTNF-R2* soluble tumor necrosis factor receptor level 2, *PTSD* post-traumatic stress disorder subjects

Mixed results on the NAC efficacy have been shown in humans with cannabis dependency (Table 2). In fact, an open-label study showed that NAC reduced self-reported marijuana use without differences in the urine cannabis toxicology in young people [61]. During randomized placebo-controlled trials in young people with cannabis dependence after 8 weeks of NAC treatment combined with behavioral therapy, the odds of negative urine toxicology were twice higher as compared to placebo [62], while NAC treatment did not change the cannabis craving in these adolescents in a secondary analysis from that clinical trial [63]. Additionally, the reduced craving was observed in both groups, suggesting that marijuana craving probably is not involved in NAC cessation effects [63]. An intent-to-treat analysis showed the relation between low pretreatment impulsivity, NAC efficacy, and negative urine toxicology for cannabinoids [64]. On the contrary, another clinical trial involving 12-week NAC therapy in adults with cannabis use disorder, did not show differences in cannabis abstinence and urine cannabinoid tests compared to the placebo-treated group [65]. However, the analysis of a subgroup of adults with co-occurring depressive symptoms and cannabis dependence found an association between higher baseline depression and decreased abstinence rates during the trial, and neither NAC nor placebo attenuated the depressive symptoms [66]. It should be emphasized that while subgroup analyses can be suggestive, they do not give strong evidence that the drug actually helped to a specific subgroup. Taken together, the co-occurrence of the behavioral therapy and NAC treatment seems to contribute to decreased cannabis use in individuals, however, further studies are required to clarify these beneficial effects.

Despite the fact that several preclinical studies confirmed the beneficial effects of NAC treatment on nicotine reward, the clinical studies are not that convincing (Table 2). Healthy individuals with NUD treated with NAC reported a reduction in the number of cigarettes smoked, however, there were no differences in craving, withdrawal, and biochemical verification of smoking [67]. Three-and-a-half day treatment with NAC (3.6 g daily) during short-term abstinence in heavy smokers did not evoke significant effects on craving and withdrawal symptoms; however, individuals treated with NAC experienced less pleasure from smoking the first cigarette posttreatment [68]. On the contrary, the positive effect of NAC at a lower dose (2.4 g daily) during 3.5-day monetary-incentivized smoking abstinence on nicotine abstinence, craving, and resting-state functional connectivity in frontostriatal areas was presented [69]. Four-week co-administration of varenicline (an α4β2 nicotinic receptor antagonist) and NAC reduced the number of cigarettes per day in adult smokers, however, a low abstinence score at the study end was presented [70]. In patients with co-occurring NUD and gambling, NAC treatment with augmented behavioral therapy during the first 6 weeks was beneficial but returned to baseline in the 3-month follow-up [71]. Eight-week NAC administration did not change daily cigarettes smoked and withdrawal [72]. Twelve-week NAC treatment at a dose of 3 g daily evoked a reduction in the number of cigarettes smoked, exhaled CO, and depression severity [73]. Adjunctive NAC at lower doses (1.8 g daily) significantly reduced abstinence score and inflammation state (soluble tumor necrosis factor receptor 2 levels) without the effect on anxiety and depression, as well as on the metabolism components [74]. Additionally, a larger study consisting of 8 and 16 weeks NAC treatment (1.8 g daily) with a 42-week post-discontinuation follow-up has been started [75]. In conclusion, a recommendation for NAC use for NUD cannot be made at this time, as further research is required.

The effects of NAC on cocaine-seeking behavior have been well studied in animal studies. In healthy cocaine-dependents, 4-day NAC treatment reduced the withdrawal symptoms and craving [76]. A follow-up study showed that NAC administered during two 3-day inpatient hospitalizations separated by 4 days reduced the desire for cocaine use induced by drug-related cues, as well as decreased interest and time for a view cue [77]. At the same time, 4-day NAC treatment reduced craving and the motivational qualities of a cocaine challenge injection but did not affect euphoric properties in individuals with CUD [78]. In an open-label study, a reduction in the self-reported cocaine use was reported in NAC-treated cocaine-dependent patients after 4-week treatment [79]. Higher glutamate levels in the dorsal anterior cingulate cortex associated with higher impulsivity were detected in patients with CUD compared to healthy controls, that were reduced after a single administration of 2.4 g NAC [16]. It should be noted that increased glutamate levels are typical during cocaine relapse, what suggests that NAC may have potentially positive effects in CUD [5, 80]. Moreover, 25-day NAC treatment increased cognitive control without the effect on working memory in cocaine-using men [81]. Eight-week NAC treatment did not reduce cocaine use in patients with CUD, however, it was shown that NAC prevented cocaine relapse rather than promoted initial drug-abstinence [82]. Attenuation of cocaine-cue attentional bias and reduced intranasal cocaine self-administration were observed after NAC treatment [83] (Table 2). It should be mentioned that according to the latest systematic review and meta-analysis performed by Duailibi et al., NAC was superior to placebo in diminishing craving symptoms in SUD [84]. However, this meta-analysis was based on seven randomized control trials with heterogeneous methodology and a small sample size (*n* = 245) and further studies are necessary to determine the potential impact of NAC on craving symptoms in SUD.

Eight-week co-administration of NAC and naltrexone in adults with methamphetamine use disorder did not affect cravings, drug use, urine toxicology, depression, anxiety, disability, and quality of life at different points of the study, compared to the placebo group [85]. On the contrary, another clinical study showed that 8-week NAC administration reduced methamphetamine craving [86] (Table 2). However, further investigation in a larger population is required to demonstrate the exact NAC efficacy.

## Summary

This review provides proof for NAC efficacy in SUD in animal models (Fig. 1). As far as beneficial effects of NAC are concerned, the modulation of GLT-1 seems to be crucial for diminishing the excitatory glutamatergic transmission observed after drugs of abuse. However, the efficacy of NAC in different drug dependencies has not been established yet. It should be emphasized that in preclinical studies the experimental conditions are stable, where no other sources of variance exist beyond the experimental variables and even a small effect of NAC may be detected using animal models. On the other hand, in human studies, more factors are implicated and the clinical efficacy is more difficult to investigate. NAC is safe and well-tolerated, but the most likely outcome, even with more studies, larger samples, and better designs, is that NAC will be a small add-on to usual treatment and/or psychotherapy for SUD. While preclinical studies have shown promise, further clinical studies and trials concerning the exact effects of NAC on SUD will be required before including this drug in general clinical practice and to point to potential groups that can actually benefit from the drug.

## Abbreviations

AUD: Alcohol use disorder
CUD: Cocaine use disorder
GLT-1: Glutamate transporter 1
GSH: Glutathione
NAC: N-acetylcysteine
NUD: Nicotine use disorder
SUD: Substance use disorder
System x_c_^−^: Cystine/glutamate antiporter
xCT: Catalytic subunit of system x_c_^−^
